# Skin Tone Estimation under Diverse Lighting Conditions

**DOI:** 10.3390/jimaging10050109

**Published:** 2024-04-30

**Authors:** Success K. Mbatha, Marthinus J. Booysen, Rensu P. Theart

**Affiliations:** 1 Department of E&E, Stellenbosch University, Stellenbosch 7602, South Africa; katlego.mbatha@gmail.com; 2 Department of Industrial Engineering, Stellenbosch University, Stellenbosch 7602, South Africa

**Keywords:** CNN, skin tone estimation, skin tone classification, machine learning, lighting conditions, Monk skin tone

## Abstract

Knowledge of a person’s level of skin pigmentation, or so-called “skin tone”, has proven to be an important building block in improving the performance and fairness of various applications that rely on computer vision. These include medical diagnosis of skin conditions, cosmetic and skincare support, and face recognition, especially for darker skin tones. However, the perception of skin tone, whether by the human eye or by an optoelectronic sensor, uses the reflection of light from the skin. The source of this light, or illumination, affects the skin tone that is perceived. This study aims to refine and assess a convolutional neural network-based skin tone estimation model that provides consistent accuracy across different skin tones under various lighting conditions. The 10-point Monk Skin Tone Scale was used to represent the skin tone spectrum. A dataset of 21,375 images was captured from volunteers across the pigmentation spectrum. Experimental results show that a regression model outperforms other models, with an estimated-to-target distance of 0.5. Using a threshold estimated-to-target skin tone distance of 2 for all lights results in average accuracy values of 85.45% and 97.16%. With the Monk Skin Tone Scale segmented into three groups, the lighter exhibits strong accuracy, the middle displays lower accuracy, and the dark falls between the two. The overall skin tone estimation achieves average error distances in the LAB space of 16.40±20.62.

## 1. Introduction

The accurate estimation of human skin tone from colour images is important in diverse applications that rely on precise colour representation in visual data. This research topic is closely intertwined with the pursuit of colour accuracy and colour constancy, affecting technologies such as facial recognition [1,2,3], medical imaging [4,5,6], and the cosmetic and beauty industry [7]. In these domains, the quality and correctness of outcomes are intrinsically tied to the faithful recovery of an individual’s skin tone. The results of failing to do so lead to sub-optimal performance, erroneous results, and sometimes, a complete breakdown in system functionality.

In the medical field, for instance, the diagnosis of skin diseases heavily relies on the analysis of skin colour variations. The cosmetics industry similarly hinges upon accurate skin colour measurement, enabling precise recommendations for foundation shades or the creation of personalised cosmetics tailored to an individual’s unique complexion [8,9,10,11,12,13].

However, perceived colours and tones in the skin are a manifestation of not just the pigmentation and skin properties but also the source of the light that is reflected by the skin. Within the scope of this study, we explore and investigate some underlying causes of inaccurate colours in images captured through various light sources, focusing on the following factors:

- Skin Tone Effects: Inherent properties governing how light interacts with an individual’s skin, particularly skin reflectance, impact the camera’s ability to capture dynamic range and colour representation, emphasising the importance of a deeper understanding for precise skin tone estimation [14,15].
- Light Source Variability: The challenges in achieving uniform skin tone estimation arise from colour discrepancies caused by the type and placement of light sources, presenting a considerable challenge due to the ever-changing lighting conditions in the real world [16,17]. Predominantly, these factors are intensity and colour spectrum.

Approaches based on computer vision technologies tend to use machine learning to address these challenges. In the realm of deep machine learning methodologies such as Convolutional Neural Networks (CNNs), effective data processing is crucial. CNNs exhibit the potential to address data-related challenges in skin tone estimation by revealing concealed data patterns. However, the efficacy of CNN models in this context heavily relies on the quality and diversity of the training data. Biased or inaccurate datasets can impede the model’s ability to generalise across various skin tones and conditions, impacting the accuracy of skin tone estimation. The goal is to contribute to advancements in fields reliant on accurate colour representation, facilitating improved applications in facial recognition, medical diagnostics, security systems, and cosmetics [18,19].

The challenge in developing such approaches is not limited to the method and CNN model but largely depends on the dataset used to train and assess it.

### 1.1. Related Works

Various works from the literature have tried to address this problem. We highlight some of their objectives, methods, and datasets.

Kips et al. [20] developed a skin tone classifier for online makeup product shopping. Sobham et al. [4] developed a method to assess the progress of wound healing, which has had a substantial impact on treatment decisions.

Borza et al., Lin et al., and Newton et al. used standard CNN architectures in their studies, achieving notable accuracy rates without providing detailed training parameter information. Borza et al. employed the VGG-19 architecture, achieving accuracy rates of 94.10%, 98.68%, and 82.89% for dark, light, and medium skin tones, respectively. Lin experimented with VGG-16, VGG-19, ResNet18, ResNet34, and ResNet50, reporting accuracy rates ranging from 71.29% to 83.96%. Newton et al. used a DenseNet201 model pre-trained on the ImageNet dataset for skin lesion classification, achieving accuracy rates of 98.10% on the ISIC2018 dataset and 91.20% on the SD-136 dataset.

In contrast, Kips et al. and Sobham et al. developed custom CNN models. Kips et al. adapted the LeNet model from the ResNet architecture for skin tone estimation into LAB colours, revealing specific hyperparameters, such as an AdaMax optimiser with a learning rate of $5\times 10^{-4}$ and a β1 value of 0.9. They applied data augmentation by randomly flipping images horizontally, training on both colour-corrected and non-colour-corrected images, with reported errors of 3.91 ± 2.53 and 4.23 ± 2.72, respectively. Sobham et al.’s custom CNN model featured four conditional layers and a fully connected layer, with fine-tuned hyperparameters achieved through the scipy library’s techniques. The Bayesian optimisation technique yielded the best performance, reaching an accuracy of 84.50%. Optimal hyperparameters included 128, 256, 512, and 512 feature maps for Conv-1 to Conv-4, dropout with 0% probability, Sigmoid activation functions, a learning rate of 0.0001, and a weight decay of 0.001.

#### 1.1.1. Skin Tone Scales

Kips et al., Newton et al., and M. Sobham et al. employed the standard Fitzpatrick Skin Tone Scale to classify their image data. In contrast, Borza et al. categorised their facial images into three manual labels—dark, medium, and light—for their eyeglass recommendation project. Lin used a four-point non-standard skin tone scale: sallow, dark, pallid, and red.

#### 1.1.2. Datasets

Both Kips et al. and Sobham et al. manually collected their datasets. Kips et al. used a diverse group of participants representing various skin tones in the United States for skin colour measurements from three facial regions. These measurements were averaged to establish a ground-truth skin colour. Participants took makeup-free photos while holding a colour calibration target and used their personal smartphones for image variation. Different lighting conditions, such as tungsten, fluorescent, outdoor, and indoor, were used for image capture. Manual annotation of colour calibration target positions ensured accuracy, with 2795 images from 655 participants used for model training.

In contrast, Sobham et al. captured colour images from 65 video frames at four subject locations under three lighting conditions: yellow, white, and ambient light. This dataset included images from five subjects, and each image was labelled with the Fitzpatrick Skin Type (FST) as a reference for skin characterisation.

Borza et al. developed a method to aid with the selection of eyeglasses inspired by recent fashion trends, where choosing the right eyeglass frame is crucial. The selection process involved various factors, such as face shape, skin tone, and eye colour, which can be challenging and costly when conducted by human analysts [7]. The training dataset was created by combining facial images from various face databases, including the Caltech [21] dataset, the Chicago Face dataset [22], the Minear–Park database, and the Brazilian face database, with the categorisation based on subjects’ skin colours.

Lin [5] developed an approach to accurately classify Chinese skin tones for disease diagnosis. The author did not specify the source of the data.

Newton et al. [6] proposed a method to detect skin lesions and examined the impact of varying skin tones on detection accuracy. They used the ISIC2018 and SD-198 datasets, both of which are key for skin disease analysis. ISIC2018 contains 10,015 dermoscopic images across seven classes. Meanwhile, SD-198 comprises 6584 clinical images representing 198 skin diseases with 17 classes.

#### 1.1.3. Data Pre-Processing Methods

All researchers considered various pre-processing steps, with cropping and scaling being common practices. Kips et al. employed cropping to isolate the face and subsequently scaled the images to a size of 128 × 128 × 3 for their non-standard LeNet model. On the other hand, Lin aimed to train both VGG and ResNet models. He began by cropping faces and scaling the images to dimensions of 224 × 224 × 3 and 227 × 227 × 3. Following this, he applied gamma correction, resulting in images of varying shades. Sobham et al. also applied cropping; however, their paper used images captured from different body parts rather than facial images. Consequently, patches of skin were collected and scaled to a size of 28 × 28 × 3.

Conversely, Borza et al. compared two methods for skin tone classification. The first involved an SVM classifier, where skin tone patches were extracted from the face. Each patch was then converted to RGB, HSV, LAB, and YCrCb colour spaces, and these images were concatenated into a long feature vector. Subsequently, the dimensionality was reduced using Principal Component Analysis (PCA). Their second model was a CNN, where from the images, faces were cropped, and the resulting images were scaled by 40% horizontally and vertically. Their final images were of size 224 × 224 × 3.

In the literature, researchers take diverse approaches influenced by factors such as data type, pre-processing methods, skin tone scale, and CNN model choice. Given the absence of a universally correct method, outcomes depend on best practices and experimental efforts within the CNN technology domain, leading to varied insights.

Public datasets prove valuable for CNN model training, addressing the challenge of data collection. However, certain prior studies [5,7] predominantly featured images under diverse lighting conditions, with limited variation in skin tones. This consideration prompts the authors of this project to explore data collection methods, drawing inspiration from [20] for image acquisition as a foundational framework.

Key aspects identified from the literature include the absence of a universally favoured scale for skin tone classification, with the choice depending on the desired granularity. Additionally, the practice of cropping and scaling the facial region for model specifications is crucial in pre-processing methods. This project aims to incorporate these techniques and employ well-defined accuracy assessment metrics specific to the model types developed. Inspired by past research [6], standard architectures like VGG and ResNet, pre-trained models, and hyperparameter tuning from [4] will be integral components of this project’s investigation.

### 1.2. Research Gaps and Contributions

Previous research on skin tone estimation has often overlooked the crucial aspect of consistent performance across diverse skin tones, leading to biases, such as the under-representation of darker skin tones. Additionally, widely used datasets like ISIC2018, SD-198, and others lack specific designs for skin tone information, limiting their applicability. For example, Lin’s focus solely on Chinese skin variations further restricts the global perspective on skin tone diversity.

This paper addresses these limitations by curating a more balanced dataset that spans the entire spectrum of skin tones. Another significant gap is the absence of standardised protocols for data structuring, CNN model optimisation, and effective training for skin tone estimation. This paper contributes to the existing body of work by comprehensively evaluating various model setups, aiming to establish best practices and identify areas for improvement in future research on this critical topic.

**Figure 1:**
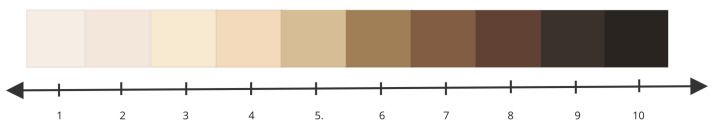
Monk Skin Tone Scale indexes used in this study.

In our model, the output is a single decimal value representing a Monk skin tone colour index within the range of 1 to 10, with a margin of error of ±0.5 units. Figure 1 shows the mapping of these indices to the Monk skin tone colours. The RGB colour space distances between the Monk skin tone colours are non-uniform, averaging 41.25 units. The linear relationship assumption between indices was made for simplicity during data collection.

## 2. Methodology

This section first describes the data-capturing process, followed by the development of the CNN model. These two components comprise the bulk of the experimental setup. However, some nuances in the setup for the individual experiments are described in Section 4.

### 2.1. Data Gathering and Dataset Creation

#### 2.1.1. Output Dataset Requirements

In this section, the requirements for the dataset are outlined, delineating the essential conditions that the dataset must meet for training and validating a CNN-based skin tone classifier model.

The skin tone distribution in the dataset should ideally maintain an equal number of images across all colour classes according to the Monk Skin Tone Scale, thereby avoiding skin tone bias and enhancing the model’s impartiality [23]. To ensure practicality in data collection, the dataset must encompass common light types encountered by individuals, including fluorescent, LED, and halogen [24], along with variations in light intensity for each type.

To cater to potential future research needs, participants were mandated to rotate their heads in five specified directions for all 15 light types, encompassing front-facing, left-facing (35°), right-facing (35°), upward-facing (35°), and downward-facing (35°) positions [25]. The dataset’s magnitude must guarantee the effective training and generalisation of the neural network model, with a minimum benchmark of 2750 images per specific head rotation. Considering image capture across 15 lighting conditions for each head rotation, a minimum of 183 individuals must participate in data collection, emphasising that a larger dataset size confers advantages for enhancing model performance [20,26]. Furthermore, to facilitate testing and validation with colour-corrected image data, all captured images must incorporate a reference colour chart, crucial for computing colour histograms and applying colour correction to the images if deemed necessary.

#### 2.1.2. Room Setup and Configuration

**Figure 2:**
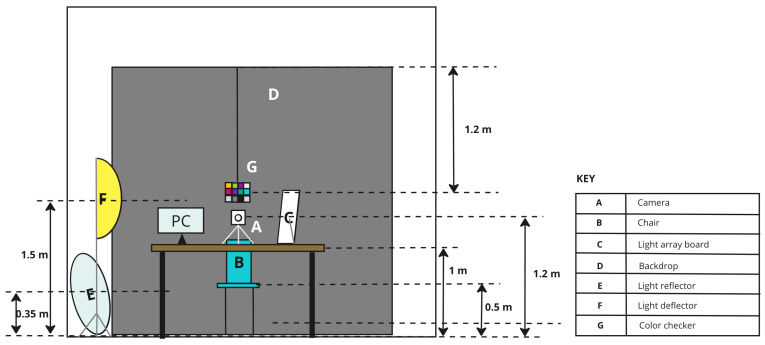
Image studio setup viewed from the front.

**Figure 3:**
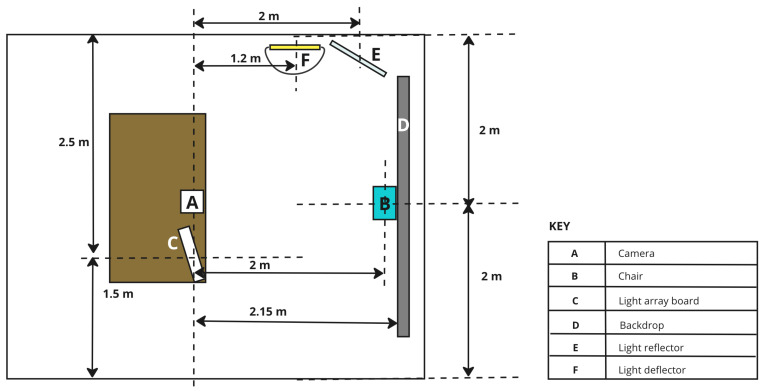
Image studio setup viewed from the top.

**Figure 4:**
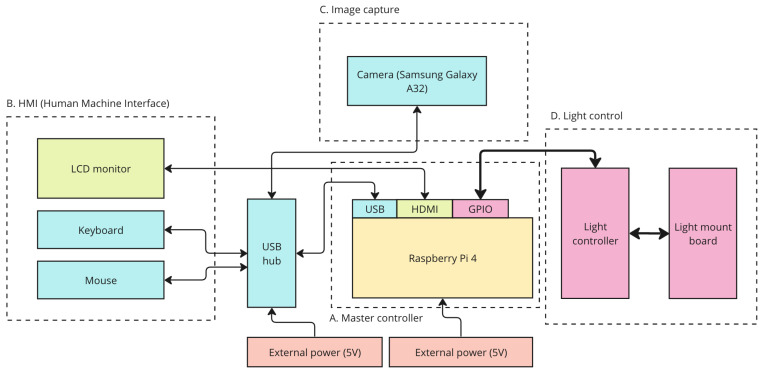
Image capture electronics system block diagram, showing all peripherals and their interfaces.

A studio space was arranged for capturing participant images, ensuring precise control over various factors such as equipment selection, spatial arrangement, and ambient light conditions. The setup aimed to create a controlled environment, and the equipment arrangement is illustrated in Figure 2 and Figure 3. The equipment comprised a Samsung Galaxy A32 smartphone used for image capture, configured with predefined settings, including a white balance of 3500 K, exposure value of −0.5, shutter speed set to 1/350, and ISO set at 800. Additionally, the equipment list encompassed a circular silver reflector, a light deflector, a backdrop in grey, a chair equipped with floor markers to ensure consistent participant positioning, a reference colour chart suspended from the top bar of the backdrop, a skin tone lookup chart used for manual label assignment, and a data logging system with a backup PC. The entire image-capturing system, including the hardware, software, and mechanical components, was automated and managed using a Raspberry Pi 4. The diagram for this system is shown in Figure 4.

The light sources, mounted on a custom-made wooden light array board, included fluorescent (warm and cool white), LED (warm and cool white), and halogen lights, each with distinct spectral properties captured by a high-precision spectrometer. The light arrangement aimed to minimise visible head shadows and maintain consistency in lighting across different head rotations. This study also employed a skin tone lookup chart, reference colour chart, and manual skin tone labelling (by the participant, assistant, and researcher) to enhance accuracy in skin tone assignments. Overall, the detailed equipment and setup ensured a controlled environment for systematic data collection.

#### 2.1.3. Dataset

Following a month-long data collection campaign at Stellenbosch University in South Africa, we successfully enrolled and obtained images from participants representing a diverse range of skin tones. The total count of individuals who participated exceeded the minimum requirement outlined in Section 2.1.1, which was 183. The resultant dataset possessed distinct characteristics: a total of 285 unique individuals, comprising 21,375 images. Each identity was associated with 15 different lighting conditions, each captured with five different head rotations, resulting in 75 images per identity.

#### 2.1.4. Skin Tone Distribution

**Figure 5:**
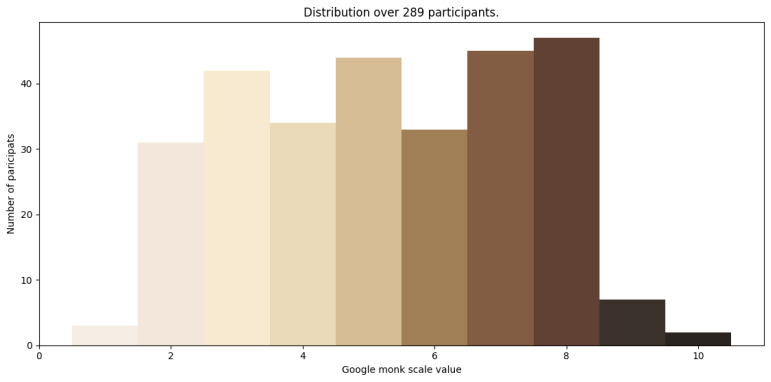
Dataset’s skin tone distribution.

Figure 5 illustrates the distribution of skin tones within the image dataset. In the case of Stellenbosch, South Africa, where the dataset collection campaign took place, it proved more challenging to recruit individuals at the extreme ends of the skin tone spectrum, such as those close to Monk skin tones 1 and 10, resulting in a relatively lower representation of data for these skin tone categories.

#### 2.1.5. Justification for Regression Approach with the Monk Skin Tone Scale

We recognise that the Monk Skin Tone (MST) Scale is not inherently a linear model since it was designed to take the non-linear human perception of colour into account. The decision was made to leverage the ability of regression models to capture the non-linear continuous nature of skin tone perception, as we view skin tone as better represented by a continuous value rather than discrete classes.

### 2.2. CNN Model Setup and Configuration

#### 2.2.1. Data Pre-Processing

This project’s emphasis on facial skin tone involved automated face detection techniques for cropping facial regions from images, focusing on front-facing views to comprehensively capture facial structures. The resulting dataset of 4275 front-facing images underwent eye detection-based cropping to address alignment issues, with images resized to 224 × 224 pixels for compatibility with the VGG architecture. A slight background allowance was included for potential model performance benefits. Generated using scripts from this project’s repository [27], the final dataset adopted a 65:35 training-to-validation partition ratio to ensure exclusive representation of specific identities in either set, preventing biased results in the validation set and allowing for a dedicated assessment of skin tone [28]. Due to the dataset’s size, further partitioning into a test set was considered impractical; instead, it was decided to rely on a substantial validation set. K-fold cross-validation was avoided due to potential bias with limited samples [29].

The partitioning of the dataset into training and validation sets was carried out using a pseudo-random number generator, employing the Python library random, which possesses a linear distribution. This resulted in the data distribution for each class in both sets being fairly balanced. However, any missing data points or imbalances were addressed through regularisation and the Mean Squared Error (MSE) loss function. This enabled the simplification of the fit by interpolating the existing points [30,31]. It was also expected to facilitate the model’s better convergence towards data points that were not well represented, such as Monk skin tones 1, 9, and 10, as observed in Figure 5.

Additionally, regularisation techniques, i.e., data augmentation, including horizontal and vertical flipping, quadrupled the dataset’s size, enhancing model generalisation, a crucial factor for CNN performance.

#### 2.2.2. CNN Architectures and Design Decisions

For this project, the VGG-16 architecture was chosen among various CNN architectures due to its prevalent use in skin tone estimation research, the availability of pre-trained weights on facial image data, and its compatibility with the VGGFace dataset’s pre-trained model weights. The selected deep learning framework, PyTorch, aligned with the facial recognition model used in a related paper [32], streamlining integration into this project’s code [5,7,33,34]. Experiments were conducted on a Dell Precision 3650 desktop computer with an NVIDIA GeForce RTX 3090 GPU. The base model, inspired by VGG-16 with modifications in the output layers, was transformed into a regression model, producing a single decimal value. Convolutional layers incorporated batch normalisation and ReLu activation functions, while fully connected layers featured Sigmoid activation and dropout with a probability of 0.5. Expansions of the layers included additional BN layers, ReLu and Sigmoid activation functions, and dropout. Time constraints limited the exploration of the impact of removing BN and pooling layers from the original VGG-16 architecture, suggesting avenues for future research.

#### 2.2.3. Use of LAB Images

Images were pre-processed by converting them from the RGB to the LAB colour space. The LAB colour space is designed to be perceptually uniform, meaning equal distances in the LAB space correspond to similar perceived colour differences by the human eye. This aligns well with the Monk Skin Tone Scale, which is also designed to be linear for human perception. Therefore, the LAB colour space is suitable for skin tone classification across diverse lighting scenarios, as its perceptual uniformity can capture subtle nuances in skin tones, and the separation of colour information from luminance is advantageous. Additionally, if L^2^ distances are calculated for error calculation, these distances would correlate well with how the human visual system perceives skin tone variations, unlike the RGB colour space.

#### 2.2.4. Hyperparameter Tuning

Table 1 outlines the hyperparameters for model training, initially set based on [20], with values tuned for optimal accuracy through a manual random search method.

#### 2.2.5. CNN Model Training Process

Throughout model training, batches of image data were fed into the model. For each batch, a vector of the estimated Monk skin tones was generated as an output. To calculate the loss between the model-estimated and target values, a vector distance between the two was calculated. The loss was determined using the Maximum Squared Error (MSE) function, as follows:

(1) $$MSE=\frac{1}{N}\sum_{i=1}^{N}{\left(\hat{x_{i}}---x_{i}\right)}^{2}$$

The loss, calculated using Equation (1), was fed back into the model using a method known as backpropagation, with the objective being to minimise it. This procedure resulted in the updating of the model’s weights, ultimately improving the model’s accuracy. In this project, the Stochastic Gradient Descent (SGD) algorithm was employed during the backpropagation process, with the learning rate (LR) and momentum values presented in Table 1.

#### 2.2.6. CNN Model Accuracy Evaluation Methods

**Accuracy Distribution over Incremental Threshold Distances**: In order to ascertain the model’s accuracy as the threshold distance between the estimated and target values was expanded, denoted as the “accuracy distribution”, the process was initiated by calculating the distances between the model’s estimated skin tones and the target skin tones, represented by the vectors $\hat{x}$ and x.

A derivation of how the “accuracy distribution” was calculated is shown in Equations (2)–(4), where $D_{\mathrm{class}}$ is a vector storing the differences between the estimated and target values, $A_{n}$ is a percentage of the number of samples ($N_{d}$) to the total number of samples with a difference less than to a constant distance *n*, and **A** is a collection of $A_{n}$s at incremental *n* values.

(2) $$D_{\mathrm{class}}=\left(\left|{\hat{x}}_{1}-x_{1}\right|,\left|{\hat{x}}_{2}-x_{2}\right|,\ldots ,\left|{\hat{x}}_{N}-x_{N}\right|\right),$$

(3) $$A_{n}=\frac{N_{d}|\left(d<n\times 0.1\right)}{N}\times 100,$$

(4) $$A=(A_{0},A_{1},A_{2},\ldots ,A_{N}),$$

**Figure 6:**
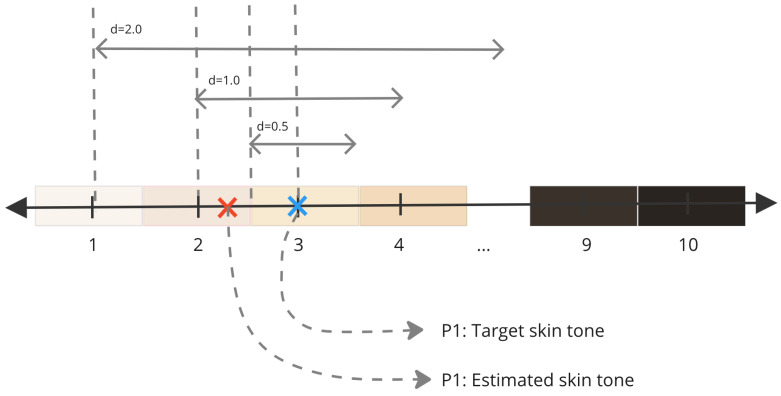
Monk Skin Tone Scale and accuracy calculations.

In Figure 6, the evaluation of a single estimated Monk skin tone value (P1) and its target (P2) is depicted, with the distances denoted as *d*. The figure illustrates that when the distance is less than 0.5 from the target, the estimated skin tone value is excluded, while inclusion occurs for distances less than 1 and 2. This calculation offers a visual representation of the proximity between the estimated and target values, aligning with the purpose of the accuracy vector. Each Monk skin tone value in Figure 6 is identified by its corresponding colour. Notably, as the distance values surpass 0.5 from the target, overlaps with adjacent skin tone classes emerge. The experiments in Section 3 and Section 4 primarily use a distance value of 0.5 as the key point for accuracy comparison.

**Average Error in the LAB Colour Space**: When evaluating skin tone estimation accuracy, the average error metric, commonly employed in prior research [20], is crucial for facilitating comparisons. Our model’s output, initially in Monk skin tone index values, needed conversion to LAB colours for this calculation. The process involved converting the estimated and target Monk skin tone values to RGB values through linear interpolation between the defined Monk skin tone colours (Equation (5)). In this equation, *C* represents the RGB Monk skin tone value corresponding to the Monk skin tone index *n*, where *n* is determined as $n=floor\left(\hat{x_{i}}\right)=\left\lfloor \hat{x_{i}}\right\rfloor$ and $\hat{x_{i}}$ represents any value from the $x_{i}$ vector.

(5) $$\hat{C_{i}}=C_{n}+\left(C_{n+1}-C_{n}\right)\cdot \left(\hat{x_{i}}---\left\lfloor \hat{x_{i}}\right\rfloor \right)$$

Subsequently, the RGB values were converted to LAB values using the Python-OpenCV cv.cvtColor method. This three-dimensional representation allowed the average error to be perceived as a combination of the mean and standard deviation calculated from the line lengths (errors) between each sample’s estimated and target values.

In the calculation process (Equations (6)–(9)), an error vector $D_{\mathrm{LAB}}$ (7) was computed by finding the distances between the estimated ($\hat{L_{i}}$) and target ($L_{i}$) LAB vectors. The average error was then determined by calculating the mean (μ) and standard deviation (σ) of $D_{\mathrm{LAB}}$.

The mean (μ) value is also known as the Maximum Absolute Error (MAE). In this paper, we present the mean and variance, resulting in the representation μ±σ. For simplicity, we refer to this metric as the “Average LAB L2 distance error”, or simply, the “Average error”.

(6) $$d_{i}=\left|\right|\hat{L_{i}}-L_{i}\left|\right|$$

(7) $$D_{\mathrm{LAB}}=\left(d_{1},d_{1},d_{1},\ldots ,d_{N}\right)$$

(8) $$\mu =\frac{1}{N}\sum_{i=1}^{N}d_{i}$$

(9) $$\sigma^{2}=\frac{1}{N}\sum_{i=1}^{N}{\left(d_{i}-\mu \right)}^{2}$$

The average error metric provides insights into the mean and standard deviations calculated from the errors between the estimated and target points for each image. In an ideal scenario of model perfection, the distances between the target and estimated values would be zero, resulting in an average error of 0±0. In the worst-case scenario, with the model performing at its worst capability, the average error would be 374.17±0. This study aims to minimise the mean and reduce the spread from the mean, where a smaller mean indicates higher accuracy and a reduced standard deviation suggests precise estimation across most images. This metric is plotted on a box and whisker plot for interpretation of the results.

## 3. Preliminary Experimental Results with Empirical Adjustments

This section presents a sequence of experimental results. First, we assess the impact of including the same identities under different light conditions in the training and/or validation datasets. Second, the best CNN model is identified, which is used to generate the main results.

### 3.1. Data Experiments: Mixed Identities and Shuffled Pixels

In this section, the impact of including images of the same identity under different lighting conditions in either the training or validation sets on validation accuracy is investigated. This involves randomly selecting images of an identity taken under various lighting conditions and distributing them within the training or validation set. Additionally, this is compared with an experiment where all images of an identity exclusively belong to either the validation or training set but not both.

**Figure 7:**
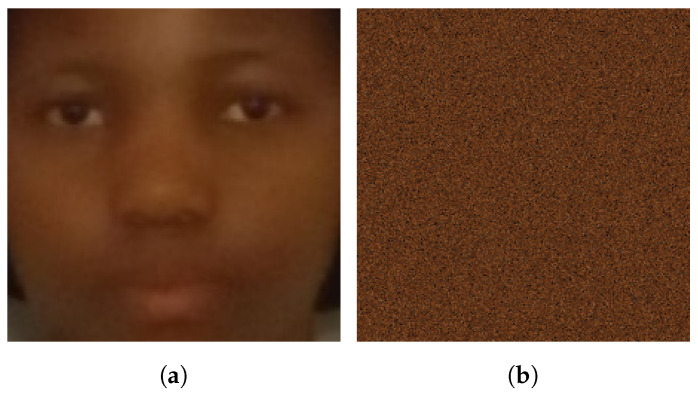
Demonstrating the effect of pixel-shuffling: (**a**) Original, and (**b**) Image with shuffled pixels.

Furthermore, the significance of an identity’s facial structures in images for skin tone estimation by the CNN model is explored. This exploration may involve the implicit detection of skin regions on the face or considering them as a colour reference. This is contrasted with an experiment in which facial structures in the images are altered by randomly shuffling pixel data. Ultimately, the dataset configuration that produces the best results, as determined by the outcomes, is used in the subsequent experiments. An illustration of shuffled pixel images is provided in Figure 7.

#### 3.1.1. Data Preparation

Four dataset permutations are created:

- **Mixed identities dataset**—This includes images of the same identity with different lighting conditions in both training and validation sets.
- **Non-mixed identities dataset**—This includes images of the same identity exclusively in either the training or validation set.
- **Mixed identities and shuffled pixels dataset**—This is a mixed identities dataset with randomly shuffled pixel data.
- **Non-mixed identities and shuffled pixels dataset**—This is a non-mixed identities dataset with randomly shuffled pixel data.

#### 3.1.2. Model Design and Training Parameters

The regression base model and training parameters from Section 2.2.2 and Section 2.2.4 are used.

#### 3.1.3. Model Validation Results

The validation results for all dataset permutations are summarised in Table 2. The accuracy is the percentage of images with a scored distance ≤ 0.5 from the target class.

#### 3.1.4. Discussion

**Mixed identities dataset**—The findings presented in Table 2 indicate that while this dataset configuration achieved the highest validation accuracy, it did not yield the best training accuracy. Notably, the validation accuracy surpassed the training accuracy, which can be explained by the presence of unlearned versus learned lighting conditions within the dataset. Since this attribute is not controlled but randomised, it can impact the model’s performance. However, this dataset configuration was not used in the subsequent experiments because in real-world scenarios, the identities present in the test dataset are typically unavailable in the inference data.

**Non-mixed identities dataset**—For this dataset configuration, the validation accuracy was marginally lower than the training accuracy. This outcome is a desirable behaviour and signifies effective model generalisation. The validation accuracy was the highest among all dataset configurations. Furthermore, in real-life use cases involving this system, the images present in the training dataset are not anticipated to be present in the inference dataset. Therefore, eliminating similar identity facial structures from both sets contributes to a more realistic evaluation of the system’s performance.

**Mixed identities and shuffled pixels dataset**—Within this dataset, there were no facial structures in the training or validation images, as illustrated in Figure 7. Several observations can be made based on this plot. The training and validation accuracies experienced a decline compared to the scenario where the image pixels remained unaltered. This decrease in accuracy indicates the model’s reliance on facial structures for the extraction of skin tone information from facial images. Another plausible explanation is the potential existence of an inherent relationship between skin tone and facial structures, with the model inferring such facial structures to estimate skin tone. This is akin to the observation that individuals of the same race, who frequently share a similar skin tone, often possess common facial features. This observation is a fundamental driver of research in the domains of race classification from facial images and facial recognition [1,35].

These findings underscore the significance of incorporating unaltered facial structures in images used for investigating skin tone estimation in deep learning.

**Non-mixed identities and shuffled pixels dataset**—The results for this dataset configuration are detailed in Table 2. These results reinforce the theories and conclusions discussed regarding the “mixed identities and shuffled pixels dataset”. The removal of facial structures from the data diminishes the overall accuracy of the model, leading to challenges in convergence.

In both cases where pixel shuffling was applied, the most notable feature in the images was the predominant colour, primarily the identity’s skin tone, with no other informative details to learn from. This limitation clearly demonstrates the insufficiency in addressing the problem of skin tone estimation using CNN technology.

The “non-mixed identities dataset” is deemed the most suitable for realistic results and strong model performance in the subsequent experiments.

### 3.2. Finding the Best Model

#### 3.2.1. Classification and Regression Models

In this section, the suitability of regression and classification models for skin tone classification and estimation tasks is explored, considering previous research preferences for both approaches [4,5,6,7,20]. An experiment is conducted to compare these models, utilising the same dataset and hyperparameters detailed in Section 2.2.6. The regression model, based on the setup in Section 2.1, is contrasted with a classification model.

The classification model is derived from the base model by replacing the output layer with a 10-valued probability vector/layer and then feeding it into a Softmax block. The resulting output represents the probabilities for the Monk skin tone classes, from 0 to 9, with index 0 corresponding to skin tone one. Common training hyperparameters are employed for both models. During data loading, all images are transformed from the RGB to the LAB colour space for both models, aligning with the rationale presented in Section 2.2.3.

**Figure 8:**
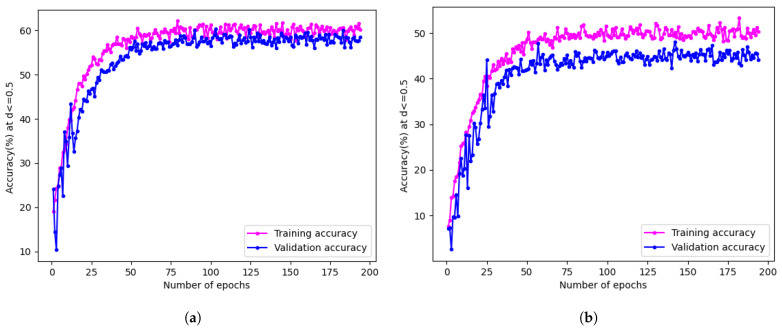
Training plots for the classification and regression models. (**a**) Training plot for the regression model. (**b**) Training plot for the classification model.

For the accuracy evaluation, the regression model follows the methods outlined in Section 2.2.6, while a different approach is adopted for the classification model due to its distinct output format. The evaluation involves calculating batch-wise accuracy and averaging the results. A comparison of the accuracy of the two models is presented in Table 3, and training accuracy plots are shown in Figure 8.

The results reveal that the regression model outperformed the classification model, achieving a higher accuracy of 58.12% compared to 46.49%. This difference can be attributed to the nature of skin tone as a continuous value, favouring regression models’ ability to capture nuances between different skin tone classes [6]. Consequently, the regression model is selected for the subsequent experiments in Section 3.2, aiming to further enhance its performance through various techniques explored in the following sections.

#### 3.2.2. Pre-Trained Regression Model

This section explores the application of transfer learning with pre-trained VGGFace weights to a Convolutional Neural Network (CNN) model for skin tone estimation. The process involves loading weights from a model trained on facial recognition tasks and freezing specific layers to capitalise on previously learned features. The VGG-16 architecture is employed, and the weights are obtained from the VGG repository. The transfer learning process includes loading weights using torch.load() and manually transferring them, with incremental freezing of layers from 0 to 12, focusing on feature extraction layers while leaving the output layers available for training.

The model used in this experiment is based on the design outlined in Section 2.2.2, and the learning parameters are consistent with those detailed in Section 2.2.4. Notably, the learning rate is reduced from $1\times 10^{-5}$ to $1\times 10^{-6}$ for fine-tuning purposes.

The validation results, depicted in the training accuracy plots in Table 4, reveal a substantial decline in both the training and validation accuracies as the number of frozen layers increases. This decline suggests that the pre-trained weights, originating from a model designed for facial recognition, did not effectively capture the nuanced features necessary for precise skin tone estimation. The divergence between the training and validation accuracies can be attributed to the dataset’s characteristics, with higher skin tone variability in the training set.

Despite various attempts, the best model only achieved 29.81% accuracy, rendering it ineffective for further exploration. This outcome underscores the inadequacy of using facial recognition weights for skin tone estimation and emphasises the importance of a dedicated skin tone dataset for improved model performance.

#### 3.2.3. Applying Colour Balancing to Image Data

This section explores the impact of light source variations during image capture on colour content and the potential improvement through colour balancing. In the data pre-processing stage, colour balancing using the Grey World Assumption [36] is integrated into the data loader, employing the colorcorrect library in Python. The regression model and training parameters outlined in Section 2.2.2 and Section 2.2.4 are used for the experiment.

**Figure 9:**
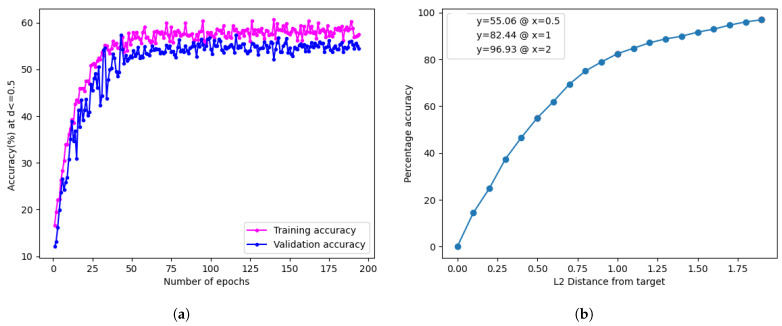
Training graph and validation results for colour-corrected data. (**a**) Training accuracy plot for the model trained on colour-balanced images. (**b**) Model accuracy distribution for the model trained on colour-balanced images.

The training accuracy plot for the model trained on colour-balanced images is presented in Figure 9a, showcasing an overall accuracy of 55.06% at an L2 distance of 0.5 from the target scores. Additionally, the cumulative accuracy distribution is illustrated in Figure 9b.

The findings suggest that implementing colour correction leads to a decrease in accuracy compared to the performance observed without applying colour correction, specifically the base model performance at 58.12%. This decline may be attributed to unintended consequences of colour balancing, such as the removal or alteration of relevant colour information for the skin tone estimation task. The process, aimed at correcting variations in lighting conditions, might inadvertently eliminate subtle variations in skin tones or textures critical for accurate classification [37].

#### 3.2.4. Using LAB, RGB, and HSV as Input Image Features

This study delves into improving the accuracy of skin tone estimation by augmenting the number of channels and incorporating additional information from diverse colour spaces. This involves combining RGB, LAB, and HSV channels during model training, inspired by relevant past research on skin tone extraction [7]. Subsequently, the model undergoes validation to assess its performance.

During the data pre-processing stage, images are transformed from RGB to RGB–HSV–LAB format. This is achieved by converting the RGB image into HSV and LAB colour spaces and then concatenating them into a single (9 × w × h) input tensor.

The model employed in this experiment is a nine-channel regression model, a modification of the model described in Section 2.2.2. It is noteworthy that the model is trained without pre-existing weights and adheres to the parameters outlined in Section 1.

**Figure 10:**
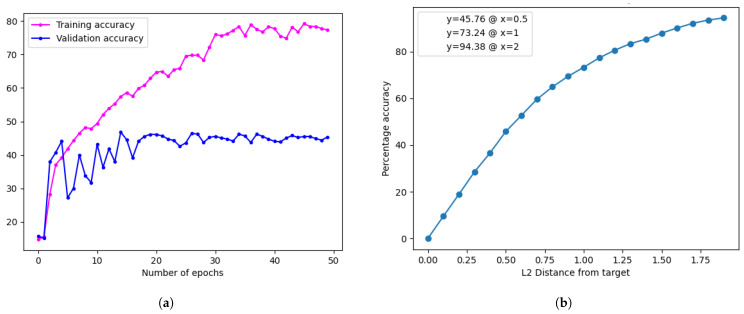
Training graph and validation results for the RGB–HSV–LAB model. (**a**) Model accuracy distribution. (**b**) Training accuracy distribution plot.

At a Monk skin distance of 0.5, the validation accuracy was 45.76%. Despite achieving a high training accuracy of 80.01%, a considerable drop in validation accuracy was observed, indicating potential overfitting, as shown in Figure 10.

This study explores regularisation techniques, such as dropout and weight decay, to address the overfitting problem. However, as detailed in Table 5, these attempts yielded consistent results, with no significant improvement in mitigating the issue.

The authors of this study acknowledge its limitations and recommend further research. Specifically, we suggest investigating the impact of enlarging the dataset size as a potential avenue for improving accuracy and resolving the observed issue.

#### 3.2.5. Summary

A summary of all experiments conducted in this section is outlined in Table 6. As per the validation results, the top-performing model was the regression model trained on the LAB images. The subsequent experiments discussed in Section 4.1 use this model.

## 4. Results

This section presents the main results of our study. First, we present the results under different lighting conditions. Second, we present the results of fine-tuning the model for these different lighting conditions. Third, we present the results of the model across skin tones on the Monk scale. Then, we discuss the model’s performance when estimating skin tones under different lighting conditions. The results are then compared with the performance of existing methods from the literature. The section concludes with a summary of the results and main findings in the context of the existing literature.

### 4.1. Experiments under Various Lighting Conditions

In this section, experiments are conducted to examine the influence of varying lighting conditions on the best-performing model identified in Section 3.2. The chosen model is subjected to two experiments: evaluating its performance with images captured under different lighting conditions and fine-tuning it on each lighting type. The subsequent discussions present the obtained results.

The first experiment focuses on assessing the model’s robustness to changing lighting conditions. The model undergoes training using the original dataset, including data from all lighting conditions detailed in Section 3.1.4. It is then evaluated with validation data specific to each distinct light source to gauge its ability to generalise accuracy across various lighting types.

For the evaluation, the dataset’s validation set is categorised into five groups based on light source types: warm-white fluorescent, cool-white fluorescent, warm-white LED, cool-white LED, and warm-white halogen. The same regression model and training parameters described in Section 3.2 and Section 2.2.4 are employed for this experiment.

**Figure 11:**
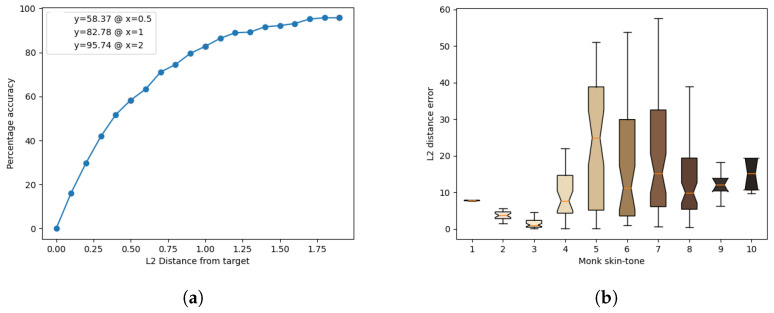
Inference results for the warm-white fluorescent light source. (**a**) Accuracy distribution. (**b**) Error for each Monk skin tone (in the LAB space).

**Figure 12:**
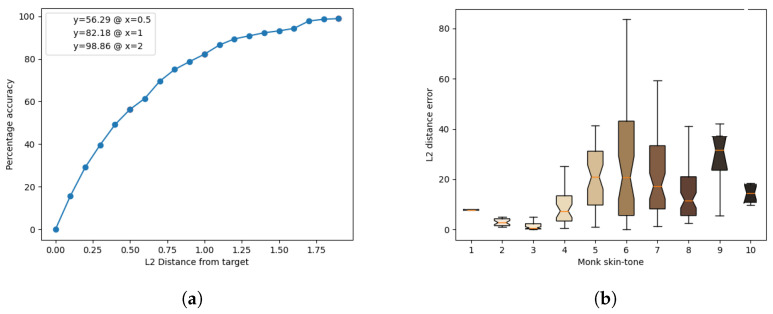
Inference results for the cool-white fluorescent light source. (**a**) Accuracy distribution. (**b**) Error for each Monk skin tone (in the LAB space).

**Figure 13:**
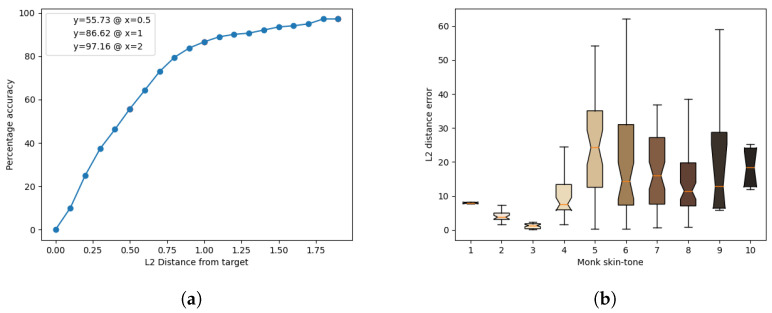
Inference results for the warm-white LED light source. (**a**) Accuracy distribution. (**b**) Error for each Monk skin tone (in the LAB space).

**Figure 14:**
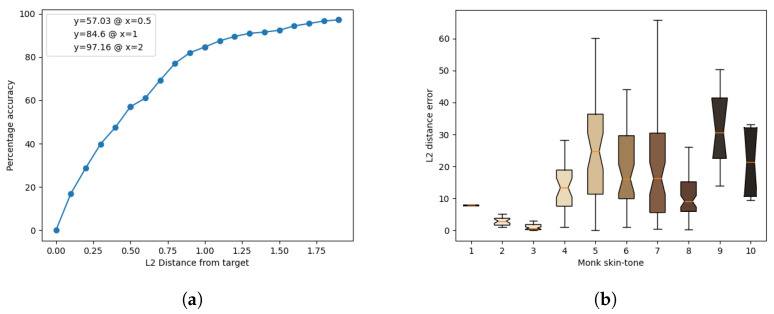
Inference results for the cool-white LED light source. (**a**) Accuracy distribution. (**b**) Error for each Monk skin tone (in the LAB space).

**Figure 15:**
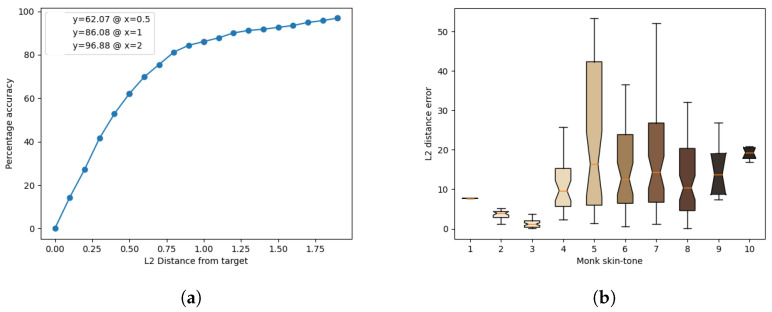
Inference results for the warm-white Halogen light source. (**a**) Accuracy distribution. (**b**) Error for each Monk skin tone (in the LAB space).

The results of the model’s inference for each light source are presented in Figure 11, Figure 12, Figure 13, Figure 14 and Figure 15. These figures include the accuracy distribution and error for each Monk skin tone in the LAB space. Subsequently, the discussion delves into a detailed analysis of the obtained results.

A comprehensive comparison of the model’s results for the different lighting types is presented in Table 7. The table includes accuracy percentages at distances of 0.5, 1, and 2 from the Monk skin tone targets, along with the average error measured in the LAB space. The data reveal interesting patterns, with warm lighting generally yielding higher accuracy compared to cool-white lighting. However, an exception is observed with warm-white LED lighting, which exhibits lower accuracy, likely due to the spectral properties of the light type.

**Figure A1:**
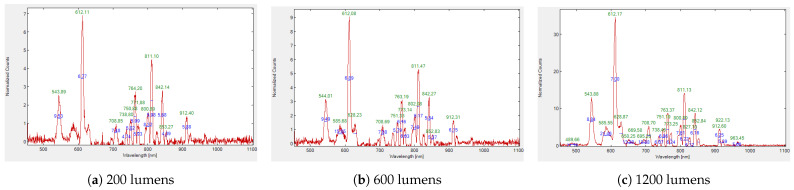
Spectral graphs for fluorescent cool-white lights with intensities of 200, 600, and 1200 lumens.

**Figure A2:**
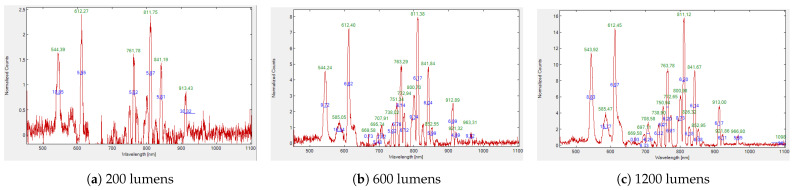
Spectral graphs for fluorescent warm-white lights with intensities of 200, 600, and 1200 lumens.

**Figure A3:**
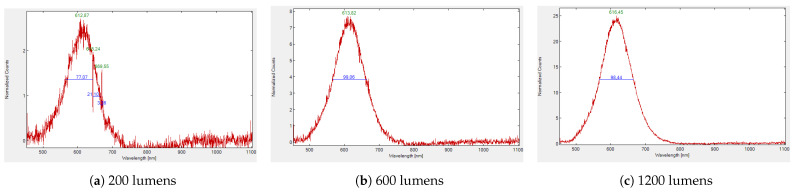
Spectral graphs for LED cool-white lights with intensities of 200, 600, and 1200 lumens.

**Figure A4:**
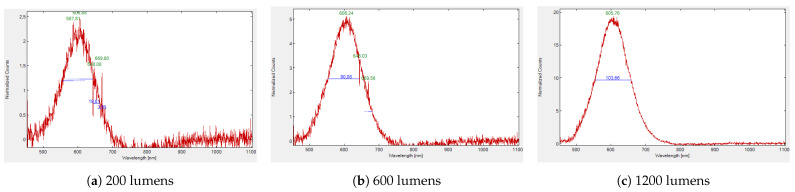
Spectral graphs for LED warm-white lights with intensities of 200, 600, and 1200 lumens.

Figure A1, Figure A2, Figure A3 and Figure A4 illustrate that warm and cool LED lights have similar spectral graphs centred around 600 nm. In contrast, fluorescent and halogen lights exhibit more spectral content in the lower wavelength range (around 300 to 700 nm for warm light) and larger wavelength range (around 700 to 900 nm for cool-white light), respectively. This difference is likely due to the use of specific combinations of phosphor light filters in LED lights. These filters modify the original light, producing distinct visual colours, unlike the light sources in fluorescent and halogen lights [38].

Analysing the results at a distance of 2 units, cool-white fluorescent lighting emerges as the top performer with an accuracy of 98.86%, while warm-white fluorescent exhibits the lowest accuracy of 95.74%. The discussion further explores the implications of these findings, including the impact of different light sources on accuracy and precision for each skin tone.

Overall, the results highlight the model’s strong performance across various lighting conditions, emphasising its generality in accurately estimating skin tone. Further details and insights specific to skin tones are provided in Section 4.3.

### 4.2. Fine-Tuning the Model on Individual Lighting Types

In the fine-tuning experiment, the regression model, initially trained with the optimal dataset configuration, underwent refinement to enhance accuracy within specific lighting scenarios. This experiment aimed to assess the model’s performance when exclusively exposed to single lighting-type data, offering insights into its adaptability to diverse lighting conditions encountered during training. The dataset was divided into five groups based on light source types (fluorescent (warm and cool), LED (warm and cool), and halogen). The regression model was fine-tuned with a reduced learning rate to prevent substantial weight updates and retain pre-trained weights, maintaining feature extraction layers in a frozen state. The results, detailed in Table 8, indicate varying impacts on the accuracy and error metrics for different light sources. While accuracy improvements can be observed for specific lighting types, decreased accuracy can be observed for others, highlighting the challenge of achieving a universally improved model through fine-tuning.

The comparison between the fine-tuned and non-fine-tuned models, based on Table 7 and Table 8, emphasises nuanced outcomes. Although overall accuracy has not significantly improved, there are notable enhancements in the average error metric for certain cool lighting types, signifying improved precision for particular skin tones. This study underscores the trade-off between optimising accuracy for specific lighting conditions and maintaining a more generalised model. Future research avenues could explore the potential differences in training a model from scratch for specific lighting conditions, providing further insights into variations in skin tone estimation under different illumination environments.

### 4.3. Model Performance Patterns across Skin Tones

**Figure 16:**
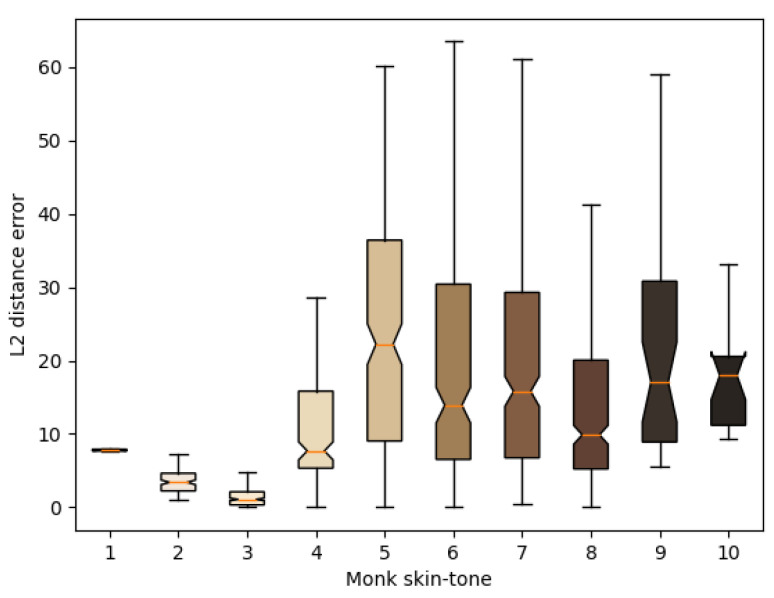
Average error for each Monk skin tone for the best-performing model.

In this section, we delve into the performance patterns of the LAB regression model across various Monk skin tones. The model’s evaluation is based on Figure 16, illustrating the average error for each skin tone. Three distinct groups emerged from the analysis:

**Group 1 (Skin Tones 1–3):** The model displayed robust performance for individuals with lower skin pigmentation, exhibiting an average error of 3.67±10.10. This can be attributed to the increased reflectance of light from light-skinned individuals during image capture, resulting in clearer facial features for the model to learn from.

**Group 2 (Skin Tones 4–7):** The model demonstrated comparatively poorer performance for individuals with mid-range pigmentation, with an average error of 22.47±23.90. Factors contributing to this include potential inaccuracies in skin tone assignments and the inherent limitations of the model.

**Group 3 (Skin Tones 8–10):** The model showed intermediate performance between Group 1 and Group 2 for individuals with higher pigmentation, with an average error of 14.52±11.58. The reduced reflected light due to higher pigmentation led to diminished visibility of facial features in the captured images.

Notably, Group 2, encompassing a broad range of skin tones, exhibited significant errors, while Groups 1 and 3, with less variability, demonstrated lower errors. The distribution of skin tones within the dataset is visually represented in Figure 5.

The observed errors may be attributed to the model’s attempt to linearly map skin tones, despite the non-linearity of the actual LAB colours. Monk skin tone distances in the LAB space reveal smaller distances associated with lower error levels and larger distances correlating with higher errors. This suggests a relationship between distance and accuracy. This study proposes exploring a non-equidistant approach for image labels based on Monk skin tone distances to enhance model performance. Further research is recommended to investigate the implications of this approach on model accuracy.

### 4.4. Influence of Light Source Variations on Skin Tone Estimation Accuracy

In Figure 11, Figure 12, Figure 13, Figure 14 and Figure 15, the average error for each Monk skin tone in the LAB space is depicted. To facilitate the discussion of these findings, the groupings outlined in Section 4.3 are applied. Table 9 provides a summary of the average errors of the regression model when tested with distinct types of light sources while being trained on all types.

Upon examining the results presented in Table 9, it is evident that the consistency of skin tone accuracy across different lighting conditions was generally not maintained for the same identity within all skin tone groups. However, it is clearly discernible that the lowest error was observed in Group 1 (comprising 399 images), with a maximum error of 5.90±20.88 for halogen warm-white lighting. Conversely, Group 2 (consisting of 960 images) exhibited the highest average error, reaching 23.74±24.40 under LED cool-white lighting, while Group 3 (including 387 images) demonstrated intermediate performance, displaying a maximum average error of 15.96±11.97 for fluorescent cool-white lighting.

Another notable observation is that when examining skin tones at the extremes, individuals with low-pigmented skin tones (Group 1) exhibited the lowest error at 2.90±3.04 under fluorescent cool-white lighting, whereas those with high-pigmented skin tones (Group 3) displayed the lowest error at 12.67±18.82 under fluorescent warm-white lighting. This leads to the conclusion that skin tone estimation accuracy for individuals with high-pigmented skin tones can be effectively obtained in warm light but is significantly compromised in cool light. Conversely, cool-white lighting enhances accuracy for individuals with low-pigmented skin tones while diminishing accuracy in warmer lighting conditions.

### 4.5. Average Error Compared to Previous Research

**Consistent Analysis of Datasets and Model Results**: In the paper titled “Beyond Colour Correction: Skin Colour Estimation In The Wild Through Deep Learning” [20], the distribution figure (Figure 4) illustrates that about 76% of participants’ skin tones, equivalent to an Individual Typology Angle (ITA) of above 20, corresponded to a Fitzpatrick skin tone of ≤3 or a Monk skin tone of ≤4.5. To ensure a fair comparison, we only compared Group 1 of our skin tone categories (as defined in Section 4.3) with the results in the aforementioned paper, as these skin tones are similar. The authors reported that the average error in accuracy for the model was 4.23±2.72, while in Section 4.3, our average error in Group 1 was calculated as 3.67±10.10. Although not a perfect one-to-one comparison, it can be inferred that our results align consistently with the authors’ findings.

**Accuracy Comparison Using a 3-Class System and a 10-Class Monk Skin Tone Scale**: In [7], a VGG-19 CNN model classified skin tones into dark, medium, and light classes. Mapping this to our 10-class Monk skin tone scale, each of the classes in the aforementioned work covers a Monk skin tone distance of 1.67. Our collective accuracy, interpolated between columns Acc. (1) and Acc. (2) in Table 7, was approximately 95%, aligning with the authors’ reported rates of 92.52% for dark, 87.69% for medium, and 94.03% for light. This confirms the challenge of distinguishing skin tones with higher class width granularity.

### 4.6. Summary

This section’s skin tone estimation experiments reveal insights into our regression model’s accuracy across diverse skin tones and lighting conditions. We examined factors like lighting types and skin tone groups, observing varying performance across lighting types and encountering challenges in the middle range of skin tones.

Notably, certain light sources yielded higher accuracy, and classifying skin tones highlighted challenges in the middle range. Discrepancies in average errors among skin tone groups underscore the complexity of capturing pigmentation variations. These results call for further research and potential enhancements in training data and methodologies.

## 5. Conclusions

This paper aimed to ensure consistent skin tone estimation across diverse conditions. An image collection campaign involved 285 participants, generating 21,375 images with a balanced representation of skin tones. Base models based on the VGG-16 architecture were introduced, with experiments emphasising identity separation and facial structures for accurate skin tone estimation. Experiments compared regression and classification models, revealing the regression model’s superior accuracy (58.12% at an estimated-to-target distance of 0.5). The pre-trained CNN models showed limitations, and colour balancing resulted in 55.05% accuracy. Increasing the dimensionality led to overfitting. The regression model with LAB images (58.12%) was chosen for further experiments. In various lighting scenarios, the model attained its peak accuracy under warm-white halogen and fluorescent lights, achieving 62.07% and 58.37%, respectively. Spectral characteristics affected the warm-white LED light, resulting in lower accuracy compared to the warm halogen and fluorescent lights. The model demonstrated good generalisation at increased estimated-to-target value distances, achieving as high as 98.86% under the cool-white fluorescent light. An analysis across Monk skin tones revealed higher accuracy for lighter tones (Group 1) and challenges with middle-range tones (Group 2). The model performed well overall, with an average error distance of 16.40±20.62 in the LAB space.

### Limitations and Future Work

While experiments with additional models, such as ResNet or Vision Transformers (ViT), could provide further insights, the primary focus of this study was to investigate the influence of different lighting conditions on skin tone estimation using a regression-based approach. Given the relatively small dataset size, ViT models may not be the most appropriate choice. Additionally, the VGG regression model demonstrated strong performance, and expanding the analysis to other architectures may detract from the core contributions of this work. Future research could explore the tradeoffs between different model types under varying lighting scenarios.

Additional suggestions for future research include verifying data labels using colour reference charts, exploring uninvestigated hyperparameters, ensuring equal skin tone distribution in datasets, incorporating other head rotations, adopting a flexible numeric scale for skin tone labels, experimenting with tightly cropped images, and assessing the model’s performance when trained only with specific lighting conditions.

This study successfully achieved its objectives, providing insights for future research in skin tone estimation.

## Figures and Tables

**Table 1:** Hyperparameter tuning initial values, deltas, and selected values.

Hyperparameter	Initial Value	±Delta	Selected/Final Value
Optimiser momentum	0.9	0.1	0.9
Optimiser learning rate	$5\times 10^{-4}$	$0.08\times 10^{-3}$	$1\times 10^{-5}$
Batch size	16	16	32
Dropout (output layer)	0	0.1	0.5

**Table 2:** Validation results for mixed identities and shuffled pixels datasets.

Experiment	Train Accuracy	Validation Accuracy
Mixed identities dataset	59.38%	66.27%
Non-mixed identities dataset	60.02%	58.12%
Mixed identities and shuffled pixels dataset	55.32%	62.53%
Non-mixed identities and shuffled pixels dataset	49.11%	52.08%

**Table 3:** Validation results comparing the performance of the regression and classification models.

Parameter	Regression Model (0.5)	Classification Model
Percentage accuracy	58.12	46.49
Average LAB L^2^ distance error	16.40±20.62	N/A ^1^

^1^ No error value since the output of a classification model is not continuous.

**Table 4:** Training and validation results for the pre-trained model.

Frozen Layers	Training Accuracy (0.5)	Validation Accuracy (0.5)
None (0)	36.85%	29.81%
Four (4)	34.85%	28.99%
Eight (8)	28.50%	23.65%
Twelve (12)	27.5%	22.70%

**Table 5:** Regularisation results for the RGB–LAB–HSV model.

Experiment	1	2	3	4	5	6
Model	VGG-16	VGG-16	VGG-11	VGG-11	VGG-16	VGG-16
Batch size	32	32	32	32	64	128
Dropout	0.5	0.5	0.6	0.6	0.8	0.8
Weight decay	0.01	0.1	0.01	0.1	0.01	0.01
Training accuracy	88.70%	77.70%	77.86%	78.36%	73.44%	72.87%
Validation accuracy	41.04%	48.02%	43.56%	44.31%	45.79%	44.59%

**Table 6:** Comparison of experiments and their respective model skin tone accuracies and average LAB L^2^ distance errors (shown as “Average error”).

Experiment	Accuracy (0.5) ^1^	Average Error ^2^
Regression model (LAB)	58.12%	16.40±20.62
Colour balanced images	55.06%	17.80±21.20
Classification model	46.49%	-
Regression model (RGB–LAB–HSV)	45.76%	18.27±21.32
Pre-trained regression model	36.85%	18.32±21.66

^1^ This denotes accuracy measurements performed at the estimated-to-target distance of 0.5. ^2^ Average error is used for the classification model because its outputs are not continuous values.

**Table 7:** Comparison of model’s results for different lighting types for accuracy and average LAB L^2^ distance error (shown as “Avg. error”).

Light Type	Acc. (0.5) ^1^	Acc. (1)	Acc. (2)	Avg. Error ^2^
Warm-white halogen	62.07%	86.08%	96.88%	16.03±21.59
Warm-white fluorescent	58.37%	82.78%	95.74%	16.25±21.64
Cool-white LED	57.03%	84.60%	97.16%	17.12±21.06
Cool-white fluorescent	56.29%	82.18%	98.86%	16.32±18.44
Warm-white LED	55.73%	86.62%	97.16%	16.40±20.30

^1^ Column names with Acc. (x) denote measurements performed at the estimated-to-target distance of “x”. ^2^ The "Avg. Error" is used for the classification model because its outputs are not continuous values.

**Table 8:** Comparison of model’s results for all lighting types at accuracy distances ≤ 0.5, 1, and 2. The respective average LAB L^2^ distance errors are shown in the “Avg. error” column.

Light Type	Acc. (0.5) ^1^	Acc. (1)	Acc. (2)	Avg. Error ^2^
Warm-white halogen	52.43%	83.18%	95.74%	18.50±23.24
Warm-white fluorescent	58.78%	82.56%	97.31%	16.41±19.66
Cool-white LED	59.77%	86.93%	96.88%	15.49±19.93
Cool-white fluorescent	57.53%	83.36%	98.01%	15.67±18.51
Warm-white LED	51.72%	83.21%	97.73%	16.86±19.69

^1^ Column names with Acc. (x) denote measurements performed at the estimated-to-target distance “x”. ^2^ The "Avg. Error" is used for the classification model because its outputs are not continuous values.

**Table 9:** Comparison of average LAB L^2^ distance errors for different lighting types and skin tone groupings.

Lighting Type	Group 1	Group 2	Group 3
Warm-white fluorescent	3.61±4.35	22.93±26.26	12.67±18.82
Cool-white fluorescent	2.90±3.04	22.08±21.13	15.96±11.97
Warm-white LED	2.99±2.85	22.38±23.98	15.49±12.12
Cool-white LED	3.16±6.07	23.74±24.40	15.16±13.05
Warm-white halogen	5.90±20.88	21.20±23.34	13.72±11.19

Group 1 refers to Monk skin tones 1–3, Group 2 refers to tones 4–7, and Group 3 refers to skin tones 8–10 (see Figure 1).

**Figure A5:**
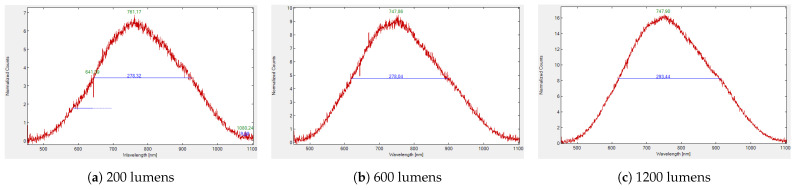
Spectral graphs for Halogen warm-white lights with intensities of 200, 600, and 1200 lumens.

## Data Availability

Data are available on request.

## Abbreviations

The following abbreviations are used in this manuscript:

CNN	Convolutional Neural Network
LAB	Lightness, A, and B colour space
HSV	Hue, Saturation, and Value colour space
RGB	Red, Green, and Blue colour space
YCrCb	Luma (Y), Chroma Red (Cr), and Chroma Blue (Cb)
ISO	International Organisation for Standardisation
FST	Fitzpatrick Skin Type
MST	Monk Skin Tone
VGG	Visual Geometry Group
SVM	Support Vector Machine
PCA	Principal Component Analysis
SGD	Stochastic Gradient Descent
LED	Light Emitting Diode
BN	Batch Normalisation
GPU	Graphics Processing Unit
ITA	Individual Typology Angle
MAE	Maximum Absolute Error
MSE	Maximum Squared Error

## Appendix A

These are the spectral graphs displaying the light spectra of all light sources utilised in this study.
